# Professor Arnold L. (Arny) Demain's historical position in the rise of industrial microbiology and biotechnology

**DOI:** 10.1093/jimb/kuab034

**Published:** 2021-06-10

**Authors:** Erick J Vandamme

**Affiliations:** Department of Biotechnology, Centre for Industrial Biotechnology and Synthetic Biology, Faculty of Bioscience Engineering, Ghent University, Ghent, Belgium

**Keywords:** Late Prof. Arnold L. Demain, Arny's Army, Impact, Industrial microbiology, Industrial biotechnology

## Abstract

This perspective text focuses on the pivotal role and historical position that the late Prof. Arnold L. (Arny) Demain has taken since the 1950s in the rise and impact of the field of industrial microbiology and biotechnology. His drive toward academic research with industrial potential—first at Merck & Co. and later at MIT—, his feeling for establishing cordial personal contacts with his students and postdocs (Arny's Army) and his ability for worldwide networking are outlined here, intertwined with the author's personal experiences and impressions. His scientific output is legendary as to research papers, comprehensive reviews, books, and lectures at conferences worldwide. Some of his research experiences in industry and academia are mentioned in a historical context as well as his relentless efforts to advocate the importance and impact of industrial microbiology and biotechnology as an essential green technology for our planet Earth.

## Introduction

This retrospective and perspective text focuses on the historical impact and pivotal position of Prof. Arnold (Arny) L. Demain (born April 26, 1927, Brooklyn, NY, USA; deceased on April 3, 2020, Madison, NJ, USA) on the development and rise of the field of industrial microbiology and biotechnology. Spanning a period of six decades, his worldwide impact on basic and applied research and applications of fermentation microbiology, industrial microbiology and biotechnology is here briefly illustrated, and it is based on and underpinned by his early and subsequent publication record. In addition to his impressive overall bibliography and great ability to establish cordial personal human relationships, he also created and nurtured social networks with his students, former collaborators and academic colleagues all over the world. Furthermore his numerous contacts and mutual interactions with the R&D and corporate biotech-sectors, especially fermentation, pharmaceutical and biotechnology companies worldwide were reciprocally beneficial to the academic world.

## “Publish or Perish” Versus “Publish … and Flourish”!

**Fig. 1:**
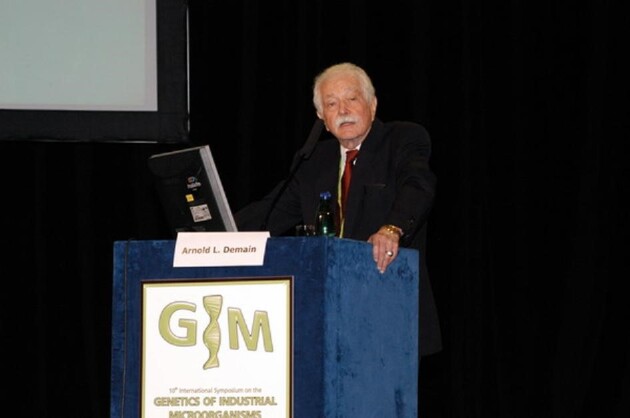
Prof. Arny Demain lecturing at the 10th International Symposium on Genetics of Industrial Microorganisms (1998), Prague, Czechia.

Over the years, Arny Demain's numerous influential early and subsequent research papers, and especially his timely reviews—with often catchy titles such as “Modern Biotechnology: no longer a minor” (Demain, 1993) or “ Small bugs, big business; the economic power of the microbe” (Demain, 2000) or “The Business of Biotechnology” (Demain, 2007)—and the many books that he (co-)edited, his inspiring lectures at MIT and at conferences worldwide as well as the national and international prizes and awards that he received did motivate and inspired many in the field to try to follow his path (Fig. 1). All these achievements made Demain's name almost synonymous with the field of industrial microbiology and biotechnology, a discipline that is now booming and expanding more than ever before. His excellent review articles were—and still are—a “must-read” and source of informative inspiration for anyone entering the field. A selection can be found in the reference list at the end of this article (Adrio & Demain, 2005, 2006; Baez-Vasquez & Demain, 2008; Baltz et al, 2017; Demain, 1959, 1968, 1971, 1972, 1973, 1974, 1978, 1993, 1998, 1999, 2000a, 2000b, 2001, 2004, 2006a, 2006b, 2007, 2010; Demain & Birnbaum, 1968; Demain & Elander, 1999; Demain et al., 1976, 1998, 2005, 2017; Demain & Fang, 2000; Demain & Sanchez, 2009; Katz & Demain, 1977; Spizek et al., 2010). He confessed in 2004 that his early mentors in the 1950s at University of California (Berkeley and Davis) and at Merck & Co. required that their students and postdocs—including him—read extensively the existing literature relevant to projects and publish a review on the state of the art, initially as introductory text of their M.Sc. or Ph.D. thesis, a practice Arny encouraged with his “followers” and … on himself for the rest of his productive life (Demain, 2004; Demain et al., 2017).

## Seeds of Arny's Drive as a Student for Academic Research in Industrial Microbiology and Biotechnology, for Cordial Networking and Corporate Relations

The official scientific career of Prof. A.L. Demain began in 1950 upon obtaining his M.Sc. degree in Microbiology with Prof. Frederick W. Fabian, a food fermentation expert, at Michigan State College (later University), East Lansing, MI, USA. His research topic was the “spoilage” (softening) of pickles during fermentation. He studied the activities of beneficial and undesirable microbes involved in the pickling process. During his undergraduate years (started in 1944), under WW II pressure, he interrupted his studies and he enlisted for 2 years (1945 and 1946) in the U.S. Navy, joining the Hospital Corps, where he received a short medical training with a mission to learn to care for dead and wounded soldiers. In the end he spent most of his military career caring for war veterans, since WW II had ended abruptly due to the use of the atomic bomb. Penicillin became just then in wider use and—being confronted with this post-war suffering, Arny must have noticed the curing effects of this wonder-fermentation product; whether this experience later activated his interest in antibiotic research is not known. During normal summer times he worked for his father's pickle factory in Chestertown, MD, buying cucumbers from farmers and shipping them by truck to the plant, stimulating his entrepreneurial and commercial skills as well. In hindsight, during his M.Sc. and Navy period, he had combined food fermentation research skills with commercial disposition and with care and empathy for people, four capabilities he obviously further developed all along his impressive career!

Arny and his wife Jody moved in the summer of 1950 for his Ph.D. studies at the Department of Microbiology (Berkeley and then Davis), University of California, CA, USA, where he was research assistant at the Department of Food Science, headed by Prof. Emil Mrak, and mentored by Prof. Herman Phaff—a pioneer of yeast biology, originally from the “Delft School of Microbiology” in The Netherlands. He obtained his Ph.D. degree in 1954 with a thesis on: “Pickle spoilage: softening by pectic enzymes, pectinase from contaminating yeasts and fungi,” where he elucidated the mechanism of pectic acid degradation by extracellular yeast polygalacturonase, an enzyme of industrial importance (Demain & Phaff,^1954^; Etchells et al., 1958; Sakai et al., 1993).

## Arny's Research Experience in an Industrial Fermentation Setting: Basic Research on β-Lactam Antibiotic Biosynthesis and on Metabolic (De)Regulation of Industrial Strains at Merck & Co.

In 1954, after his academic start, he was hired by the pharmaceutical company Merck & Co., Inc., as a researcher, first at their penicillin factory in Danville, PA. There he became fascinated and inspired by Jackson W. Foster's book “The chemical activities of fungi” to delve into the mechanisms of the penicillin fermentation (Foster, 1949). J.W. Foster, then a professor at University of Texas, had been a student of Nobel Laureate Selman Waksman at Rutgers University and had been working before at Merck on penicillin fermentation. Arny did basic research in a small Microbiology Group, while his colleagues were involved in applied research such as strain improvement and pilot plant fermentation development. He pioneered the use of starved and washed resting microbial (fungal) cell suspensions to carry out their enzymatic reactions in chemically defined solutions incapable to support growth, a technique he had learned while at Berkeley (Demain, 2004). He found that antimetabolites of cysteine (*S*-ethylcysteine) and of valine (α-methylvaline) inhibited penicillin biosynthesis by *Penicillium chrysogenum*, in the presence of the side chain precursor phenylacetate, and that the inhibition was reversed by adding the two natural l-amino acids, despite d-valine being part of the penicillin nucleus. Moreover only the amino acid l-lysine was found to inhibit penicillin formation (Demain, 1957) and addition of l-α-aminoadipic acid—a key intermediate in the fungal lysine pathway-reversed lysine inhibition (Somerson et al., 1961). This basic research approach provided clues to the penicillin biosynthetic pathway and demonstrated that in a branched pathway leading to a primary metabolite (an amino acid) and a secondary metabolite (an antibiotic), high levels of a primary metabolite could interfere with the formation of the secondary metabolite, a first example of metabolic regulation and deregulation in (secondary) metabolism (Demain, 1959, 1968, 1971, 1973). He also found that 99% of the penicillin was excreted in the fermentation broth and that it was partially degraded during its production. In 1956, he moved within Merck to the merged Merck, Sharp & Dohme (MSD) Research Labs., in Rahway, NJ, USA, where he joined the Microbiology Department of soil microbiologist Boyd Woodruff (Woodruff, 1981). There Arny initially identified unconventional microbial growth factors such as long chain fatty acids, iron transport factors, and he devised chemically defined minimal media for example for cephalosporin C production by *Cephalosporium acremonium* (now renamed as *Acremonium chrysogenum)*, allowing detailed studies of microbial growth, precursor roles and metabolic deregulation (Demain et al., 1963).

Using auxotrophic *Bacillus subtilis* and *Corynebacterium glutamicum* mutants and antimetabolites, he also studied purine metabolism in detail to improve production of flavoring compounds such as IMP and GMP (Demain, 1978; Demain et al., 1965, 1966). In 1965, Arny was asked to form a new department that would focus on biosynthesis of the Merck fermentation product range and on mutagenesis to improve the commercial strain yields. As director of this Department of Fermentation Research at Merck & Co., he guided over 30 scientists. The department was involved in a broad range of research topics on fermentation optimization and biosynthesis of antibiotics (penicillin, streptomycin, fosfomycin) (Demain & Inamine, 1970; White et al., 1971), amino acids (monosodium glutamate, lysine, …) (Birnbaum & Demain, 1969), vitamins (B_12_, riboflavin, …) (Demain & White, 1971; Kaplan & Demain, 1970), and interferon inducers (Lago et al., 1972; Strohl et al., 2001). While at MSD in New Jersey, Arny and his group often attended seminars at the Waksman Institute of Microbiology in nearby Rutgers University, allowing cordial relations with Nobel Laureate Selman Waksman and his successors. Most of the Demain's group's basic industrial fermentation—“Merck research” was published in highly ranked scientific journals, quite unusual for the industrial setting where it all happened. It was Merck's scientific director Max Tishler who stimulated the employees “to do the quality of work that would result in scientific publications.” Indeed Merck encouraged publication after giving the legal staff 3 months to submit a patent. Arny and his team surely endorsed this view and published 60–70 papers over a time span of 15 years. In hindsight academic researchers worldwide could catch far more than just a glimpse of what was going on in a giant fermentation and drug company like Merck. This stimulated many to delve into similar research topics and build further on it for years to come, introducing the latest new genetical and molecular biotechnologies! This fruitful mutual interaction between academia and industry was to become Arny's guideline throughout his further academic career.

## Arny at MIT: International Input of Bio-Scientists and Continuous Output of Novel Bio-Science and Bio-Technologies!

**Fig. 2:**
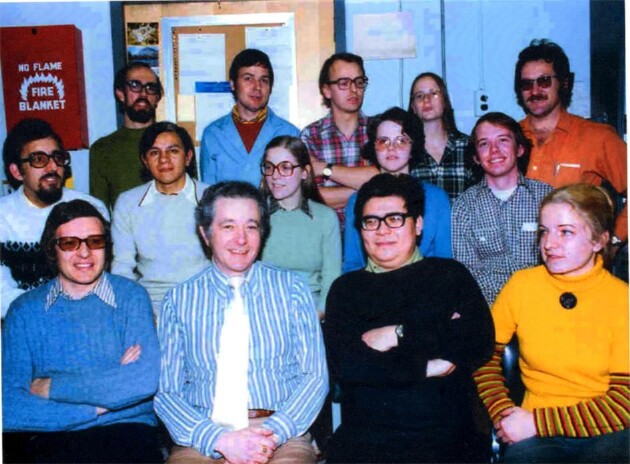
Arny's Army at MIT in 1975: Back row: Chuck Matteo (USA), Joachim Lemke (Germany), Pierre Bost (France), Cecilia Webster (USA), Yair Aharonowitz (Israel). Middle row: Juan Martin (Spain), Sergio Sanchez (Mexico), Jacqueline Piret (USA), Nadine Solomon (USA), Tom Friebel (USA). Front row: Erick J. Vandamme (Belgium), Arny Demain, Ho Coy Choke (Malaysia), Bruni Kobbe (Germany).

In 1969, Arny accepted an offered faculty position with Full Professorship in the Department of Nutrition and Food Science, at the Massachusetts Institute of Technology (MIT), Cambridge, MA, USA, and occupied that position until 2001. His preceding industrial period made him the perfect MIT professor. He was immediately involved in lecturing in MIT's famous summer course on Fermentation Technology and gradually attracted an international group of Ph.D. students and postdocs from all continents to staff his Fermentation Microbiology Laboratory. He continued his earlier Merck research on metabolic regulation of penicillin and cephalosporin biosynthesis and on amino acids using auxotrophic and idiotrophic mutants (Demain & Masurekar, 1974; Friedrich & Demain, 1977). He also resumed studies on vitamin overproduction (Demain, 1972) and on “cell free synthesis” of antibiotics, β-lactams such as penicillins and cephalosporins, thienamycin, and initiated new topics such as total cell free synthesis of antibiotic peptides such as gramicidin S and bacilysin (Demain et al., 1976; Kenig et al., 1976) (Fig. 2). His international group of M.Sc., Ph.D. students and postdocs made great progress over the next 30 years in unraveling the complex pathways, their metabolic regulation and the unusual novel enzymes involved in the producer organisms, bacteria as well as fungi. They also brought in their diverse expertise, methodologies and views, thereby broadening even more the research fields and techniques covered already at MIT. New research topics that were developed in Arny's lab in the 1980s and 1990s include use of solid state fermentation and chemostat culture, *Bacillus*-peptides and spore germination, mutational biosynthesis, use of idiotrophic mutants, molecular biology and cloning techniques, plasmid stability, structure elucidation and mode of action of mycotoxins, *Clostridium thermocellum* cellulase, bioethanol, biosynthesis and metabolic regulation of the immunosuppressant rapamycin, bioconversion of the statin compactin, formation of *Monascus* pigments, and effect of simulated microgravity on microbial growth and secondary metabolism.… A condensed overview is given in his review paper of 2004 and its literature reference list (Demain, 2004). His subsequent research publications and following comprehensive reviews—as mentioned above—made his name known worldwide to scientists doing research in fermentation, industrial microbiology, and subsequently what came to be called biotechnology. Prof. Arny Demain is now widely recognized as the “godfather” of approaches that involve applying metabolic deregulation principles, mutational biosynthesis, … (“metabolic engineering avant la lettre”) to arrive at super-producing microbial strains. He played a major role in the first stages of advocating and implementing the potential of applications of the new molecular biology for a range of industrial fermentation microorganisms. He was one of the first consultants at the very first biotechnology company, Cetus Corporation, Berkeley, CA, in the early 1970s. He never stopped emphasizing the urgent need and search for natural pharma-products and antibiotic molecules for new medical and biological targets and for novel bio-compounds with highly selective mode of action (Demain, 2004, 2006a, 2006b; Demain & Sanchez; Spizek et al., 2010). Moreover he co-edited the three editions of the “bible” of industrial microbiology and biotechnology, entitled Manual of Industrial Microbiology and Biotechnology (Baltz et al., 2010). The last edition covers all basic and bioprocess engineering aspects of the field that are essential and valuable to anyone—entering or already—involved in the field, spanning microbiologists, molecular geneticists, or bioprocess-bioengineers.

## Arny and His “Army,” Relentless Advocates of Industrial Biotechnology

While at MIT, Arny deployed—supported by an international team of (post)doctoral collaborators from all corners of the globe—a broad range of scientific achievements in the field of physiology, biochemistry, genetics, and fermentation aspects of what are now industrially very useful microorganisms, including bacteria (*Bacillus, Clostridium, Lactobacillus, Corynebacterium, Streptomyces, Pseudomonas, Escherichia coli*, …), fungi and yeasts (*Aspergillus, Penicillium, Cephalosporium, Monascus, Hansenula*, …). Overall Arny attracted, mentored and trained an international group of over 125 pre- and postdoctoral students, an “Army” of leaders in the field, who became known as “Arny's Army.”

They have followed his steps scientifically, mentally and socially in practicing and advocating the values and the uses of industrial microbiology and biotechnology to the benefit of people, our society, and the greening of our planet. The name coined for this group of former collaborators at MIT—“Arny's Army” (AA)—has become an international notion and stands for a unique community! These “next generation” students, postdocs, and collaborators are now at the helm of the field (Baltz et al., 2017; Bennett, 2017; Vandamme, 2016). Many are top scientists active in the research areas of natural products, metabolic engineering, fermentation and cell culture, bioengineering, biocatalysis, biofuels, synthetic biology, and systems biotechnology. Many members of this group have had or have now leading positions in chemical, pharmaceutical, fermentation, biotechnological, and agro- and food industries. Some members of Arny's Army have high academic or policy responsibilities and/or are head of departments of microbiology, fermentation, biochemical engineering, and biotechnology at top universities worldwide (Otero & Nielsen, 2010; Soetaert & Vandamme, 2006, 2010; Vandamme, 2016, 2020; Vandamme & Revuelta, 2016). All members of “Arny's Army” are boosters for these biotechnologies not only for other scientists and bioengineers, but also to inform the food, feed, chemical, fermentation, pharma, and other relevant and new upcoming biotech-starters and industries of its untapped potential. They continue to urge young students to enter this fascinating study and research field! Arny's scientific “offspring” has contributed a lot to many of the new “omics”—and related developments in industrially useful microorganisms, from metagenomics to proteomic analysis to directed evolution of industrial biocatalysts, metabolomics, biomathematics and biosystem analysis (Baltz et al., 2017).

**Fig. 3:**
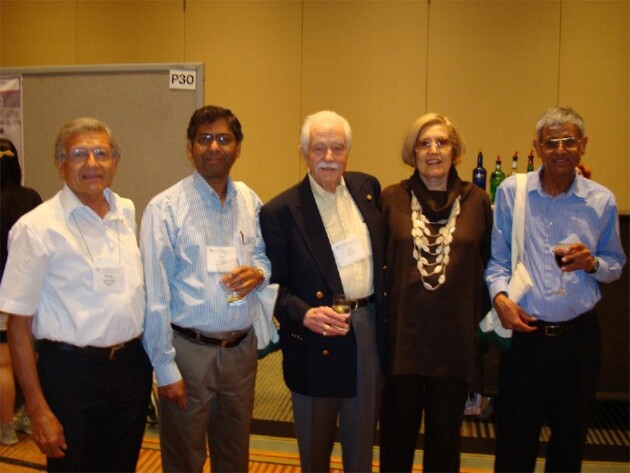
Arny at the SIM meeting in Toronto, Ontario, Canada (2009): Erick J. Vandamme (Belgium), Badal Saha (USA), Arny and Jody Demain (USA), Prakash Masurekar (USA).

Arny was active in many bio-scientific circles and received several prestigious awards, medals, and recognitions worldwide. Only a few are mentioned here: president of the Society for Industrial Microbiology (in 1990), member of the National Academies of Sciences of USA (1994), Mexico (1997), and Hungary (2002). He was on the board of governors of ASM, IUMS, … and received “Doctor honoris causa” degrees at the University of Leon (Spain), Ghent University (Belgium), Technion (Israel), Michigan State University (USA), and University of Münster (Germany). He stimulated his—former and recent—research collaborators and students to keep socially interacting and to attend seminars and scientific meetings; he recognized students and researchers qualities by introducing them to other outstanding professors in the field (Fig. 3).

**Fig. 4:**
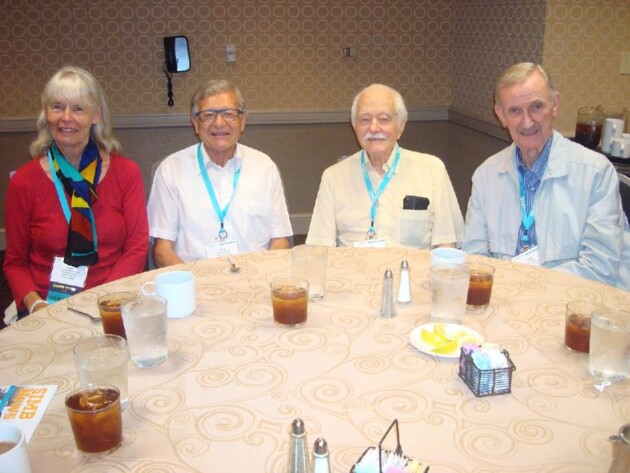
Arny Demain at SIMB meeting, New Orleans, LA, USA (2016): Kristien Mortelmans (USA), Erick J. Vandamme (Belgium), Arny Demain (USA), Herb Ward (USA).

Arny retired from MIT in 2001 and moved in 2002 to Drew University at the Charles A. Dana Research Institute for Scientists Emeriti (RISE), Madison, NJ, where he continued until 2017 with research, teaching undergraduate students and—to fulfill his passion—in writing more excellent reviews and editing journals and books (Baltz et al., 2010; Demain, 2010; Demain et al., 2017; Wall et al., 2008) (Fig. 4).

## Retrospective Comment

In overviewing the history and current state of industrial microbiology and biotechnology, the mutually beneficial relations between what is commonly called basic research and applied research is an underlying theme: about 150 years ago the largely practical investigations of Louis Pasteur and his contemporaries led to the establishment of microbiology, immunology, fermentation and biochemistry as basic sciences. From the 1945s onwards, the discovery and production-technology of antibiotics, enzymes and biochemicals, by applied microbiologists and bio-engineers provided the tools crucial to the development of molecular biology and gene technology in the 1970s. Since the 1980s, basic research in microbial and molecular genetics has delivered an array of new techniques useful to construct “tailor made” microorganisms for industrial applications. This ongoing synergy between science and technology was the underlying theme of Arny's career and it still is the key to further progress in industrial microbiology, fermentations, and microbial biotechnology. Louis Pasteur's saying of about 140 years ago remains more than ever true: “There are no such things as applied sciences, only application of sciences.” A comfort for us all is Louis Pasteur’ saying of 1882: “It is the microbes that will have the last word,” indicating in his forecast the long lasting impact of microbes and their beneficial and adverse actions and activities on people and our planet Earth, not knowing that a wide range of fermentation and biotech products would become indispensable for the health of people, for a greening of the agro-, food-, pharma-, and chemical industry and of our planet. It is fair to say that “*Arny represented a biotechnology-mind always in ferment!!”* (Demain, 2006a). We also learned from him to be “Industrious in Learning, Vigorous in Practice, Eminent in Society and Inspirational in Life!”

## Funding

None declared.

## Conflict of Interest

The authors declare no conflict of interest.
